# Buzhong Tiaogan Formula for delaying colorectal liver metastasis (liver depression spleen deficient type): A multicenter randomized controlled trial: A study protocol

**DOI:** 10.1097/MD.0000000000028040

**Published:** 2021-12-23

**Authors:** Zhi-Jie Wang, Shi-Hang Zheng, Xiao-Han Wang, Yi-Zhe Zhang, Shu-Lan Hao, Li-Kun Liu, Xi-Xing Wang

**Affiliations:** Department of Oncology, Shanxi Province Hospital of Traditional Chinese Medicine, Taiyuan, China.

**Keywords:** Buzhong Tiaogan Formula, colorectal cancer, liver metastasis, quality of life

## Abstract

**Introduction:**

Colorectal cancer has been ranked third among the most common cancers worldwide and raised to the second leading cause of cancer death with nearly one-tenth of cancer-related deaths globally, and nearly half of colorectal cancer patients present with or develop colorectal cancer liver metastasis (CRLM). Buzhong Tiaogan Formula (BTF) has been proven to treat CRLM in our team, but there are lacking of evidence on its effective in delaying colorectal liver metastasis (liver depression spleen deficiency type), so we will evaluate the efficacy and safety of BTF in preventing the occurrence of CRLM.

**Methods:**

This randomized controlled trial (RCT) will be carried out in 3 different hospitals in Shanxi Province planning to recruit 150 CRLM patients with the type of liver depression spleen deficiency. The control group will be treated by basic antitumor therapy and the treatment group will use BTF plus basic antitumor therapy. The primary outcomes will be quality of life of included patients, the time of occurrence of liver metastasis, the score of traditional Chinese medicine symptom for the type of liver depression spleen deficiency; and the secondary outcomes will include overall survival, progression-free survival, DFS, tumor microenvironment and immune state of the included patient. Safety evaluation will be recorded during the whole study. All data in this RCT will be analyzed by SPSS 23.0 software. This study has been approved by the Clinical Research Ethics Committee of Shanxi Province Hospital of Traditional Chinese medicine (2021Y-06016).

**Discussion:**

The results of this RCT will contribute to BTF for delaying colorectal liver metastasis (liver depression spleen deficient type). And the results from this RCT will be published in a relevant journal after finished.

**Trial registration:**

ChiMCTR2100005268 (September 4, 2021).

## 1 Introduction

Colorectal cancer (CRC) has been ranked third among the most common cancers worldwide and has risen to the second leading cause of cancer death with nearly one-tenth of cancer-related deaths globally.^[1,2]^ According to the latest global cancer data from International Agency for Research on Cancer (IARC), the number of new cases of CRC in 2020 was about 1.93 million worldwide and the number of deaths was up to 930,000. In China, the incidence has been jumped the second among cancer with 55 million new cases annually, and the mortality has ranked fifth among common causes of cancer death, with 28 million new deaths in 2020.^[3]^ About 20% of patients with CRC have advanced disease at the time of diagnosis, and 35% of patients treated with curative intent will develop metastatic disease.^[4,5]^

Nearly half of CRC patients present with or developed colorectal cancer liver metastasis (CRLM).^[6]^ Unfortunately, approximately 25% of CRLM are deemed to be unresectable. Chemotherapy is considered as the best strategy, which offers the best chance of cure, improving 5-year survival rates from 40% to 50%.^[7–9]^ Significant advancements in the perioperative management of CRLM have led to the ability to effectively perform more complex surgeries, such as improved diagnostic modalities, multistage hepatectomies, portal-vein embolization, and downstaging with preoperative systemic therapy.^[10]^ Despite these advances, disease recurrence is common, occurring in 60% or more of patients.^[11,12]^ But because of multiple lesions, scattered distribution, tumor location, intolerance of patients, the patients are unable to withstand accept the surgical treatment. In this pessimistic situation, other therapies should be developed. Minimally invasive treatments are the chemical treatment of the hepatic artery embolization, chemical ablation, and a variety of melting temperatures. Radiation therapies are radiation physics, radiation biology, radiation treatment technology, and equipment. Chemical and molecular targeted therapies include monoclonal antibodies, gene therapy, and biotherapy. 5-fluorouracil (5-FU) is the main chemotherapy agent in the treatment of CRC, and other drugs contain capecitabine, ticio, oxaliplatin, irinotecan. In recent years, with the progress of biotechnology, molecular targeting drugs such as Cetuximab, Panizumab and new molecular targeting drugs such as Apboxipul, Regolfinib, and Sildenib have been widely used in clinical practice. All the above treatments have certain defects and deficiencies, which can cause serious adverse reactions to the digestive system and blood system^[12]^ or damage the hepatic sinus vessels, resulting in increased perioperative bleeding, thus reducing the surgical resection rate.^[13]^

Traditional Chinese medicine (TCM) is characterized by simplicity, convenience, simplicity, and efficiency, which provides a new therapy pattern. According to a recent study, combined with Western medicine treatment or not, TCM therapies have shown efficacy for CRLM. It can not only assist the human body to restore *qi* and create surgical conditions for some CRLM patients, but also play an important role in reducing side effects from surgery, radiotherapy, and chemotherapy. Through the search of clinical and experimental studies on TCM treatment of CRLM in recent years, it is found that the treasure house of TCM has not been fully tapped in the treatment of CRLM, the research quality is low. To enrich the means of TCM treatment, it should seek more effective and more abundant methods of TCM treatment of CRLM.

Buzhong Tiangan Formula (BTF) is an experience formula in treating CRLM, which has been used for more than 20 years in our team, while there are lacking of evidence on its effective in delaying colorectal liver metastasis (liver depression spleen deficiency type). So, the purpose of this study is to verify the efficacy of BTF in preventing the occurrence of CRLM and to prolong the time phase of CRLM.

## 2 Materials and methods

### 2.1 Study design

**Figure 1:**
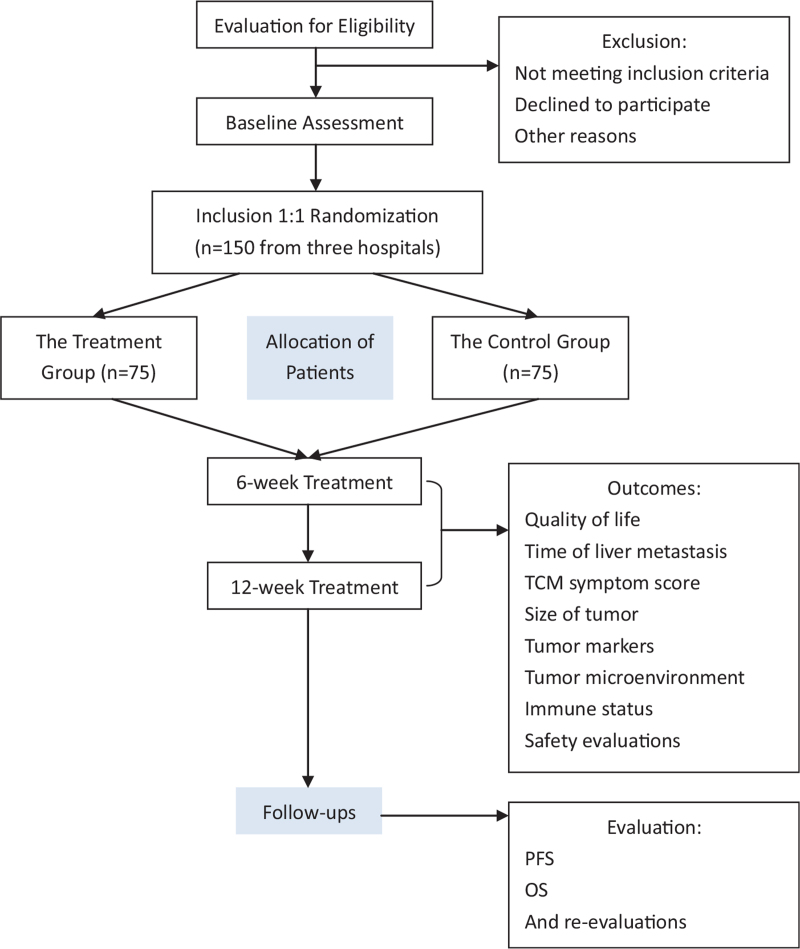
Study flowchart.

This is a prospective, multicenter randomized controlled trial (RCT) of BTD for delaying colorectal liver metastasis (liver depression spleen deficient type). Included patients are planned to be divided into 2 groups: the treatment group (received basic anti-tumor drugs plus BTD), and the control group (received basic antitumor drugs). The patients are planned to be followed ups for 2 years after 12-week treatments. The flow diagram is shown in Figure 1. This protocol followed the Standard Protocol Items: Recommendations for Interventional Trials (SPIRIT) (Fig. 1).

### 2.2 Ethics and registration

This RCT has been approved by the Human Research Ethics Committee of Shanxi Province Hospital of Traditional Chinese medicine (2021Y-06016) and has been registered under the Chinese Clinical Trial Registry (ChiCTR2100050757). The participants who must sign the informed consent form will be involved, and they can quit this study at any time.

### 2.3 Patients

#### 2.3.1 Diagnostic basis

The diagnostic criteria for CRC refer to Guidelines for diagnosis and treatment of CRC (2020); The diagnostic criteria of Chinese medicine syndrome type refer to Standard of China Association of Traditional Chinese Medicine Guideline for TCM diagnosis and Treatment of tumor (2008).

#### 2.3.2 Inclusion criteria

1. Age >18 years, excepted survival ≥6 months;
2. Patients with CRC without liver metastasis (spleen deficiency and liver depression type) that meet the above-mentioned diagnostic criteria of Western medicine and Chinese medicine and have been diagnosed by definite pathology or cytology;
3. Those who have not taken TCM and received chemotherapy in the last 1 month;
4. No other primary tumors and serious heart, liver, and kidney diseases;
5. Those who have the telephone number and address of follow-up, are willing to cooperate with follow-up, have relatively complete medical records, and have signed informed consent.

#### 2.3.3 Exclusion criteria

1. Nonprimary CRC with unclear diagnosis;
2. Unable or unwilling to receive TCM treatment;
3. The patient has developed intestinal obstruction, cannot take medication, needs intravenous high-energy nutrition, or has malabsorption syndrome or other diseases affecting gastrointestinal absorption, or has active peptic ulcer disease.

#### 2.3.4 Shedding criteria

1. Poor compliance;
2. Serious adverse events, complications, and special physiological changes should not continue to be accepted by researchers;
3. Quit voluntarily;
4. Misdiagnosed or included by mistake.

### 2.4 Sample size

According to the results from a preliminary small sample clinical trial carried out by our team and literatures, the 1-year survival rate was 65% and the median survival period was 17 months in the treatment group, and 55% and 14 months in the control group. The Ebricalc2000 software calculated that 60 cases should be required for each group, with an estimated drop-out rate of 20%, 75 participants should be enrolled in each group.

### 2.5 Randomization and blinding

The included participants will be divided into the treatment group and the control group randomly in a 1:1 ratio. SAS9.3 software will be used for generating random sequences by an independent researcher who will be not involved in the period of treatment and data analysis. The central randomization system (provided by the department of oncology, Shanxi Province Hospital of Traditional Chinese Medicine) will be used and for each center, the clinical investigators will log into the central system by telephone or internet for applying for the randomization number for a new included patient.

### 2.6 Intervention measures

The basic intervention will be basic antitumor therapies including chemotherapy, radiotherapy, immunotherapy, and targeted therapy for CRC.

The control group will just use the basic intervention, and the treatment group will use basic intervention plus BTF provided by Shanxi Province Hospital of Traditional Chinese Medicine for 12 weeks.

All included participants will be supervised by an experienced oncologist during this RCT in each center.

### 2.7 Outcomes

#### 2.7.1 Primary outcomes

1. Quality of life of CRC patients: measured by the medical outcomes study item short from health survey (SF-36) which developed by the Medical Outcomes Study and Karnofsky score (KPS). SF-36 is involving 8 aspects including physical functioning, role-physical, bodily pain, general health, vitality, social functioning, role-emotional, and mental health;
2. The time of occurrence of liver metastasis, the size of tumor, and the expression of tumor markers in the serum;
3. The score of TCM symptom for the type of liver depression spleen deficiency.

#### 2.7.2 Secondary outcomes

1. Overall survival, progression-free survival, or disease-free survival (DFS);
2. Tumor microenvironment, measured by vascular endothelial growth factor receptor (VEGFR) and Th1/Th2 in the serum;
3. Immune state of the patient, measured by subset of the class of T-cells in the serum.

### 2.8 Safety evaluation

A special safety supervisor will record each adverse event during the whole period of this RCT in detail.

### 2.9 Data management and quality control

Any changes to the protocol should be approved by the ethics committee of Shanxi Province Hospital of Traditional Chinese Medicine. All the data of patients will be recorded in case report form (CRF) by a trained researcher. All information on patients should not be shared without their permission.

### 2.10 Statistical analysis

Data analysis setting will follow intent-to-treat (ITT) principles. Data will be statistically analyzed by SPSS 23.0 software. Chi-Squared test will be used for enumeration data, and mean ± standard will be used for measurement data. Drawing survival curves of overall survival, progression-free survival, and DFS will use Kaplan–Meier and the difference between the treatment group and control group will use log-rank. If possible, subgroups analysis will be carried out by the left or right colon, gene status, sex, and age. *P* < .05 will be considered a significant statistical difference.

## 3 Discussions

In recent years, the incidence and mortality rate of CRC is rising sharply in China. CRLM has been threatening the patients without efficacy treatment with western medicine, so the important thing that needs to solve is prevention. Chinese herbal medicine has been proved that no matter herbal decoction, tablet, or patent, the herbal medicine treating CRLM is mainly based on invigorating the spleen and invigorating *qi*, combining with soothing the liver and removing blood stasis, which reflects the overall idea of TCM in treating tumors and the theory of nourishing and dispelling pathogens. And this emphasized that the treatment of metastatic tumors should be divided into general and local treatment, and that systemic treatment should be put in the first place, and local treatment is on the basis of systemic treatment.^[14]^

BTF is a formula proved by Professor Xi-Xing Wang, a famous Chinese doctor in China and composed of 15 herbals including *Huangqi, Baizhu, Shengma, Chaihu, Danggui, Huangqin, Baishao, Nvzhenzi, Wuweizi, Banzhilian, Biejia, Bayuezha, Xiakucao, Dangshen, Gancao* for treating CRLM (liver depression spleen deficiency type). Professor Wang established the therapeutic principles of harmonizing liver and spleen, reducing toxin, and eliminating disease under the guidance of the harmony method. According to the concept of “Fuzheng does not stay evil, Quxie does not hurt positive,” the herbals to nourish spleen and liver and detoxify anticancer products are used, which will ensure the patient's *qi* and blood, viscera and body in harmony, eventually pursuit the state. Only when *Yin* is at peace and Yang is compact can essence-spirit be normal to achieve the condition of “human-tumor coexistence.”^[15]^

The results from this RCT will contribute to clinical practice by BTF for delaying colorectal liver metastasis (liver depression spleen deficient type). Our goal in this RCT will be to accomplish an RCT with high quality that utilizes validated evaluation measures not only for delaying the occurrence of liver metastasis itself but also focusing on the quality of life of patients and also will explore the possible therapeutic mechanisms. And the results from this RCT will be published in a relevant journal after finished.

## Author contributions

**Conceptualization:** Shu-Lan Hao, Li-Kun Liu.

**Data curation:** Shi-Hang Zheng, Xiao-Han Wang, Yi-Zhe Zhang.

**Formal analysis:** Xiao-Han Wang.

**Investigation:** Li-Kun Liu.

**Methodology:** Shu-Lan Hao, Zhi-Jie Wang.

**Supervision:** Li-Kun Liu.

**Validation:** Yi-Zhe Zhang.

**Writing – original draft:** Zhi-Jie Wang, Shi-Hang Zheng.

**Writing – review & editing:** Zhi-Jie Wang, Yi-Zhe Zhang, Li-Kun Liu, Shi-Hang Zheng, Xiao-Han Wang.
